# Mental wellbeing during pregnancy and the transition to motherhood: an explorative study through the lens of healthcare professionals

**DOI:** 10.1186/s12884-025-07894-5

**Published:** 2025-08-01

**Authors:** Katarina Ekelöf, Gerd Almquist Tangen, Christine Delisle Nyström, Marie Löf, Kristin Thomas

**Affiliations:** 1 Department of Medicine, Karolinska Institute, Huddinge, Sweden; 2 Department of Pediatrics, Halland Hospital Halmstad, Halmstad, Sweden; 3 Department of Pediatrics, Institute of Clinical Sciences, Sahlgrenska Academy, University of Gothenburg, Gothenburg, Sweden; 4 Department of Medical and Health Sciences, Linköping University, Linköping, Sweden

**Keywords:** Maternity healthcare, Mental health, Mental health promotion, Mental wellbeing, Perinatal period, Pregnancy, Resilience

## Abstract

**Background:**

The perinatal period can be an overwhelming time involving significant physiological and psychosocial changes. The perinatal period has shown to be a time of increased vulnerability for onset or relapse of mental health problems (e.g., depression, anxiety). Mental wellbeing such as resilience have positive effects on women’s physical and mental health during pregnancy as well as on pregnancy related outcomes. However, more knowledge is needed on perinatal mental wellbeing and how it can be promoted. This qualitative study explores mental wellbeing during the perinatal period and promoting factors through the lens of healthcare professionals.

**Methods:**

Individual interviews were conducted with healthcare professionals (*n* = 16) through the chain of care from pregnancy to post-partum (i.e., maternity healthcare, maternity wards and child healthcare) and included midwives, obstetricians, psychologists and child healthcare nurses. A semi-structured interview guide and open-ended questions were used, and inductive content analysis was performed.

**Results:**

In analysis, an overarching theme: “The being and the becoming of a mother – equanimity in the transition to motherhood” emerged to describe mental wellbeing during the perinatal period. Three main categories were found to describe factors promoting mental wellbeing: (i) Inner resources (e.g., trusting the process of pregnancy), (ii) Experiencing trust during the transition (e.g., having someone to share experiences with), and (iii) Having access to a caring and supportive network (e.g., emotional support). These factors were described by professionals as being important for mental wellbeing as they help women during their transition to motherhood.

**Conclusion:**

Mental wellbeing during the transition to motherhood can be promoted by supporting and building resources for equanimity. Findings emphasize the need to target mental health promoting factors on multiple levels including strengthening the inner resources of the individual but also building social support structures around the individual.

**Supplementary Information:**

The online version contains supplementary material available at 10.1186/s12884-025-07894-5.

## Background

The perinatal period is a time of increased vulnerability for the onset or relapse of mental illness. A recent case control study showed that 7% of pregnant women screened positive for mental illness at the first antenatal visit with the majority (85%) having at least one mental disorder or risk factor for a mental disorder such as depression, anxiety or severe fear of childbirth [1]. Indeed, pregnancy has been described as an intense developmental phase similar to adolescence and has been termed matrescence [2]. Many women regard pregnancy and the post-partum period as a positive time although common sources of reported distress include changes in physical appearance and comparison to other women, loss of sense of self, possible impact of distress itself on the developing foetus and concerns about not bonding with their baby [3]. In addition, the COVID-19 pandemic further contributed to perinatal mental health problems (due to social isolation and added health worries) [4] with long COVID‑19 also being a prevalent condition among pregnant women today [5].

Mental health problems during pregnancy can have severe and long-lasting consequences for pregnant women, the infant, and their partners [6]. A systematic review including twenty population-based studies showed an association between untreated antenatal depression and adverse infant outcomes such as increased rates of operative delivery, premature birth, small for gestational weight and infant neurodevelopmental outcomes [7].

Mental health includes mental illness and mental wellbeing [8]. Mental wellbeing includes realising one’s abilities, coping with stressors in life, working productively and fruitfully and contributing to one’s community [9]. Research has shown that by enhancing mental wellbeing, symptoms of mental health problems (e.g., depression) can be reduced and increased function can be achieved by building resilience to challenges in life [10]. Thus, by promoting mental wellbeing one may reduce the pathological symptoms and limit suffering [11].

Overall, research on maternal mental wellbeing emphasises the importance of its promotion during pregnancy and that the positive effects are independent of mental health problems such as worry and anxiety. For instance, maternal mental wellbeing during pregnancy has been found to uniquely correlate with higher birth weight and gestational length [12] as well as positive cognitive and emotional development of the offspring up to 24 months after birth [13]. Furthermore, in an observational study psychological wellbeing has been recognised as a protective factor against the occurrence of pre- and post-partum depression [14]. However, reports show that maternity healthcare centres struggle to prioritise the promotion of mental wellbeing (rather than treatment of illness per se) due to limited knowledge, confidence and time [15, 16]. Thus, we need to explore what entails mental wellbeing during pregnancy and develop cost-effective ways for its promotion.

Pilot studies testing the feasibility of mental health promotion targeting pregnant women have been shown to promote both mental wellbeing and reduce symptoms of mental illness [17–19], but studies are difficult to compare due to different intervention content and format. Specifically, interventions can vary between physical activity [20–22], mindfulness [19, 23] and multi-component positive psychology programs which combine different exercises and methods to increase positive thoughts, behaviours, and emotions [17]. Interventions also differ regarding delivery and duration e.g., some interventions are offered during the perinatal period, and others during pregnancy [23–25] or postpartum [26]. Additionally, delivery format differs between in-person group sessions [23, 27] or individual approaches using digital resources such as telephone-delivered interventions [26], web-based or mobile phone-based interventions [24, 25]. Furthermore, outcome measurements for mental health during pregnancy differ extensively between mental illness and mental wellbeing, with limited consensus on outcome measurements for mental health during pregnancy. Also, studies on mental wellbeing during the perinatal period are often based on women’s subjective experience and emotional wellbeing concerning their experiences of physical changes during pregnancy and of labour and birth as well as attitudes and beliefs about self-conception, confidence and self-efficacy [28–32].

The present study employs a health care professional perspective along the entire chain of care during the perinatal period which adds to current understanding of mental wellbeing. Health care professionals may offer an “out-sider” perspective on mental wellbeing and in their reflections, draw from clinical experience among a diverse group of women contributing to rich data. This study is part of a larger research project that aims to develop and evaluate a digital intervention delivered through maternity healthcare centres promoting mental wellbeing during the perinatal period. This interview study describes the initial explorative work carried out, which will be used in intervention development. The specific aim of this interview study was to explore mental wellbeing during the perinatal period and its promoting factors, from a professional healthcare perspective.

## Methods

### Design

A qualitative exploratory design study using an inductive approach was chosen to explore the phenomenon of mental wellbeing during pregnancy and after birth including factors promoting maternal mental wellbeing. Inductive content analysis, as defined by Elo and Kyngäs [33] was used to describe and explore variations in health professionals’ experiences to provide a rich understanding of mental wellbeing [34]. The consolidated criteria for reporting qualitative research checklist [35] was used to ensure correct and adequate reporting of the study (see supplementary material 1).

### Study setting

**Fig. 1:**
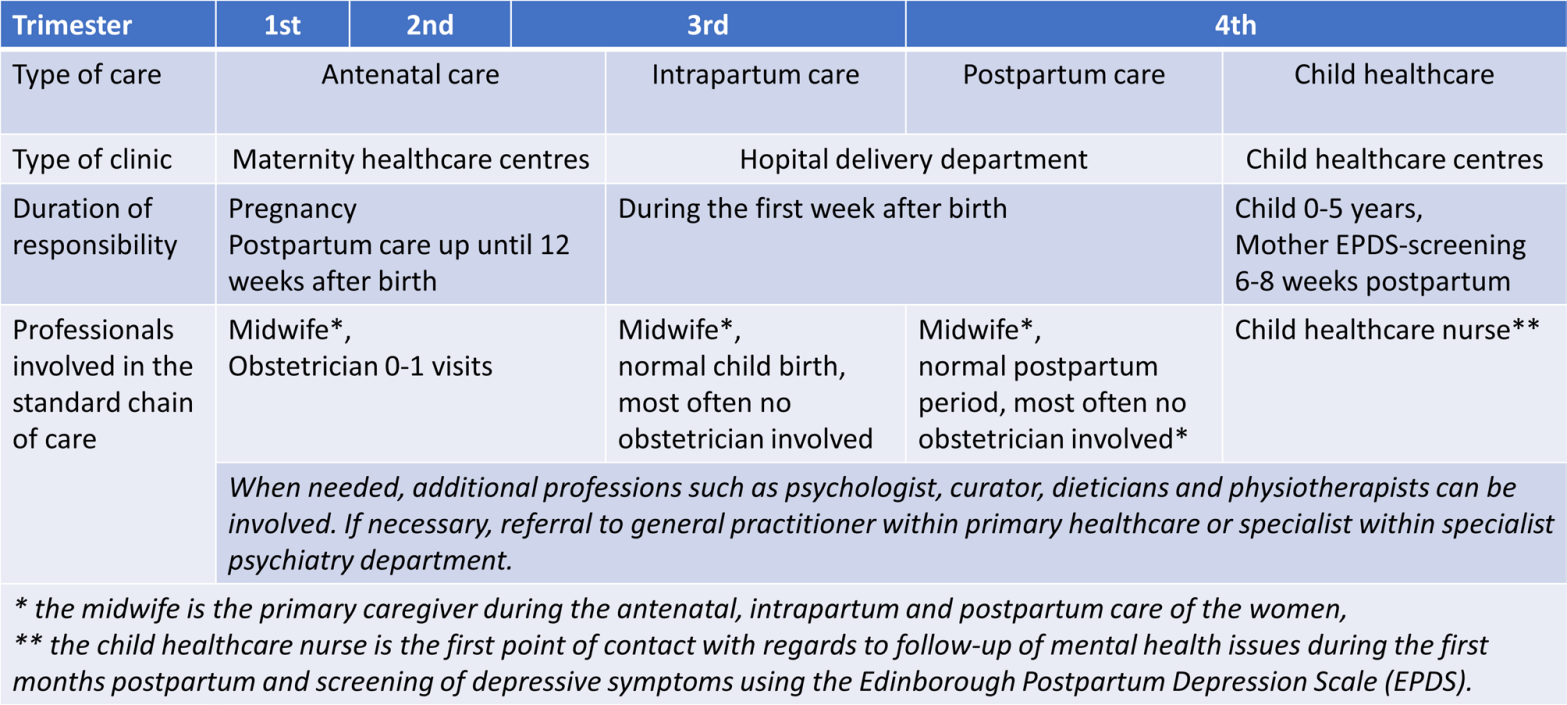
The chain of care for women during the perinatal period in Sweden as adapted from Barimani et al., [36]

This study included health care professionals working with pregnant women and new mothers throughout the chain of care. In Sweden, the chain of care during the perinatal period includes antenatal and postpartum care at maternity healthcare centres, hospital maternity wards (delivery units) and child healthcare centres for follow-up care as previously described by Barimani et al. (see Fig. 1) [36]. Specifically, different levels of care and professionals are involved throughout pregnancy, childbirth and the postpartum period, depending on psychological and physical complications. Figure 1 outlines the type of care, type of clinic, duration of responsibility as well as the professionals involved in the basic healthcare programme. Pregnancy is divided into three trimesters and an additional fourth trimester has been added to illustrate the continued attachment and dependence on nurturing during the first three months after birth, as well as the prevalence of postpartum depression [37]. A total of five regions in the south of Sweden took part in this study (Halland, Skåne, Stockholm, Värmland and Västra Götaland) encompassing both rural and urban areas and approximately 58 thousand births each year. Specifically, clinical professionals working with women during pregnancy and postpartum within maternity healthcare centres, hospital delivery departments and child healthcare centres were recruited. Professions identified to be able to provide knowledge of perinatal mental health were (i) midwives in maternity healthcare centres and delivery units being the main point of contact for pregnant women, (ii) psychologists working within maternity care and child healthcare and being the point of contact for mental health care, (iii) obstetricians with a placement within maternity health care and delivery units and (iv) child healthcare nurses which are the main point of contact for new mothers and responsible for screening for depressive symptoms postpartum.

### Informants and recruitment

A purposeful snowball sampling method was utilized to recruit health care professionals with extensive expertise and experience working with mental health promotion among pregnant women and new mothers. The sampling method strived to find informants with significant knowledge about mental wellbeing during the perinatal period and its promoting factors, that is, pregnancy and post-partum. Initially, managers working at Maternity Healthcare Centres, Hospital delivery units and child health care centres within five regions were contacted for the approval to identify and invite eligible individuals for interviews. The managers dispersed information about the study to their staff and direct contact was taken by mail and phone with the informants that showed interest or was suggested as experts. Snowball recruitment was also employed to recruit informants [34, 38]. Specifically, individuals, in their role as an expert on maternal mental health, were asked after the interview if they knew anybody else eligible to take part in an interview. Throughout recruitment, purposive sampling was employed based on inclusion criteria (i) currently working as a midwife, psychologist, obstetrician or child health care nurse at at a maternity healthcare centre, hospital delivery unit or child health care centre with and (ii) have at least two years’ clinical experience within women’s health care, hospital delivery or child healthcare. All informants were sent written study information prior to the interviews and provided their informed consent before the interview commenced.

### Data collection

The interviews were performed between October 2021 and March 2022 primarily via video link (one in-person interview). The first author conducted all the interviews (KE, female PhD student with no clinical experience working with mental health in the pregnant population). A semi-structured interview guide was used, and probing questions were added to aid informants in reflection and elaboration. As a first step, a pilot interview was conducted with a research colleague to test the interview guide. This interview was not included in the research material and did not result in any significant changes to the interview guide.

Before each interview commenced, informants were allowed to ask any questions and verbal informed consent to take part in the study and the interview was gathered. The interviewer (KE, female PhD student) recorded her preconceptions about mental wellbeing during pregnancy e.g., expectations, assumptions and experiences before the first interview. The interviewer introduced herself as a PhD student without any previous clinical experience within perinatal mental health. The interviews were audio recorded and transcribed verbatim, and field notes were taken during each interview. After 14 interviews, we perceived that no new data emerged during the interviews. However, at this point, two additional interviews were performed to ensure data saturation [39].

### Data analysis

The analysis was carried out using inductive content analysis according to Elo and Kyngäs [33]. During the preparation phase, first author (KE: female PhD student) and co-authors (GAT and KT: female senior researchers experienced in qualitative methods) documented their preconceptions about mental wellbeing during pregnancy e.g., expectations, assumptions and experiences. Potential assumptions or biases in the analysis were thus predominantly addressed through self-reflection but also through investigator triangulation. Then, the transcripts of the interviews were read by each author (KE, GAT and KT) to gain a sense of the data and the dataset as a whole. Unit of analysis was identified by the first author (KE) for all the transcripts and by authors (GAT, KT) for the first five transcripts. All data (e.g., sentences, shorter paragraphs) that were deemed to elucidate perceptions of mental wellbeing during pregnancy and promoting factors were selected as units of analysis.

Then, in the organising phase, words and phrases that were considered units of analysis were coded (open coding) by the first author (KE) into coding sheets in the software program NVivo (released 2022). In addition, authors GAT and KT performed open coding on the first five transcripts to contribute to investigator triangulation and enable iterative discussions throughout. Then, the first author (KE) made a first draft of sub-categories (grouping) by analysing differences and similarities between open codes. Preliminary sub-categories were discussed between authors (KE, GAT and KT) until agreement was reached regarding labels and grouping (categorization). In a final step, potential similarities of the sub-categories were discussed and compared (KE, GAT and KT), and sub-categories similar in content were grouped into generic categories (abstraction). In addition, an overarching theme was created which was interpreted to illustrate both generic categories and sub-categories.

## Results

In total, 16 informants (14 women) were interviewed. The interviews were on average 53 min, with a range of 33 to 72 min. Midwives and obstetricians worked at maternal healthcare centres and hospital delivery units; psychologists at maternal healthcare centres and/or child healthcare centres and child healthcare nurses at child healthcare centres. The informants had a long (median of 18.5 years) experience of working with women during the perinatal period, handling a wide range of women from high and low sociodemographic backgrounds. All informants had clinical experience in working with mental health and could reflect on the mental wellbeing of women during the perinatal period. In addition. several informants had clinical experience of working with mental illness such as fear of childbirth and postpartum depression. Table 1 includes further details on the informant’s characteristics.

**Table 1:** Characteristics of informants

Profession per clinic	Number of informants
Midwives	
Maternity healthcare	4
Delivery ward	1
Psychologists	
* Maternity healthcare and/or * *child healthcare*	5
Obstetricians	
* Maternity healthcare and/or hospital delivery ward*	3
Child healthcare nurses	
* Primary child health care*	3
**Clinical experience**	**Median years (range)**
In profession	21 (6–45)
In the profession and working with pregnant women	18.5 (2–25)
In the profession and working with new mothers	20.5 (2–25)

**Fig. 2:**
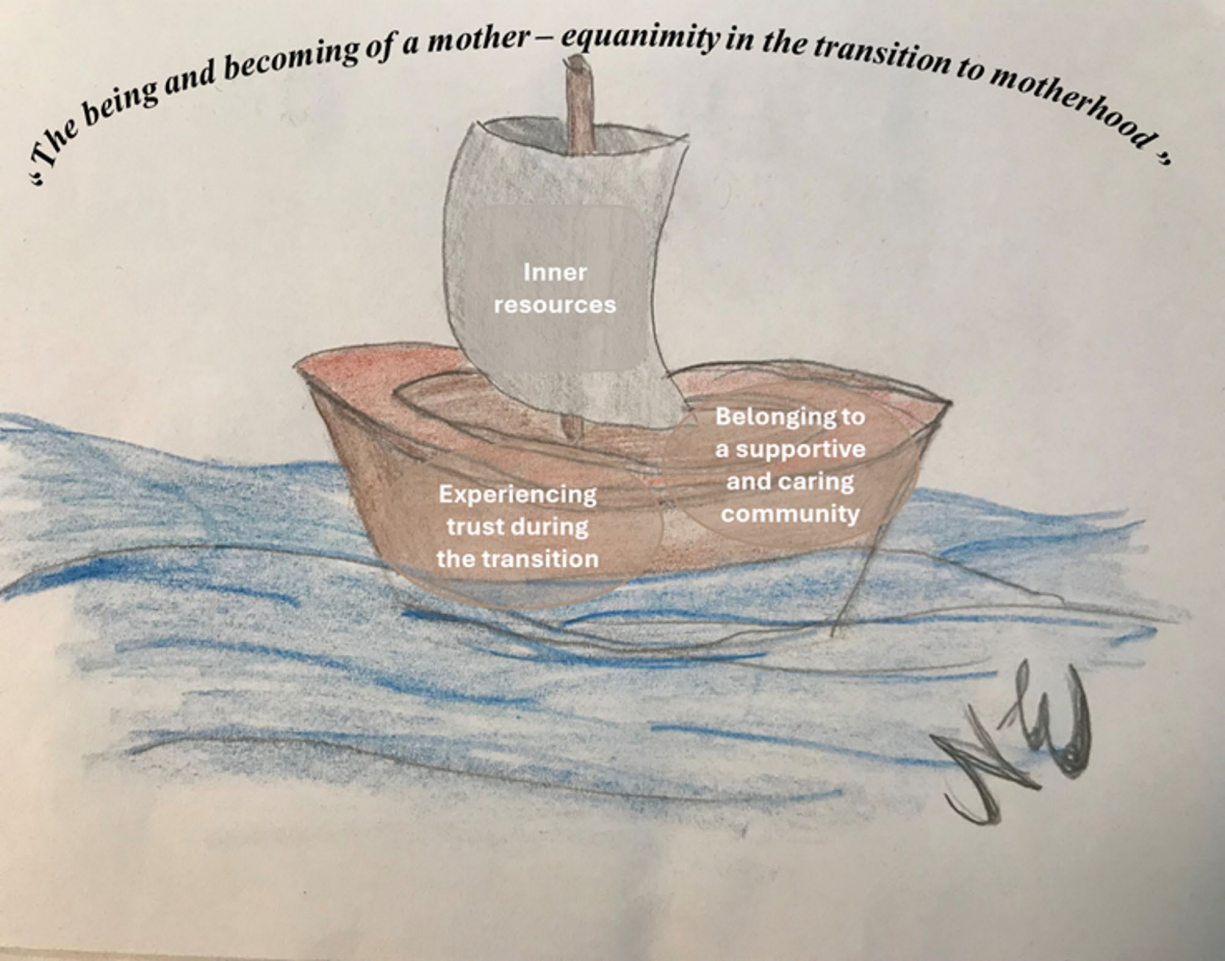
An illustration of the overarching theme “The being and becoming of a mother– equanimity in the transition to motherhood”. The sea symbolising the ever-changing and potentially challenging transition to motherhood. Factors contributing to equanimity (inner resources, experiencing trust, and belonging to a supportive and caring community) in the transition to motherhood are depicted as a boat (illustrated by Nova Ekelöf)

An overarching theme describing healthcare professionals’ perceptions of mental wellbeing during pregnancy and early motherhood emerged: “*The being and becoming of a mother – equanimity in the transition to motherhood”.* The healthcare professionals in the study described mental wellbeing as striving towards equanimity (balance) during a life-changing period (Fig. 2). They described the duality of pregnancy, being one; but also, about to be two. The duality includes individual self-growth in terms of developing and adapting to a new role and becoming a mother as well as developing an attachment to the growing life within and to the future child. The transition to motherhood was described by professionals as a learning phase through which the pregnant woman tries to find an approach to motherhood, an inner compass that guides through the many changes that occur during pregnancy. Mental wellbeing during the transition was described as curiosity about the baby and the future.

Three main categories were found to describe factors promoting mental wellbeing: (i) inner resources, (ii) experiencing trust during the transition, and (iii) having access to a caring and supportive network. These factors were described by professionals as being important for mental wellbeing as they help the transition to motherhood. The main categories and the sub-categories are illustrated in Table 2 and are described below. The data analysis process is described in the supplementary material (Supplementary material 1: Examples of the data analysis process from unit of analysis to main category).

**Table 2:** Overview of the overarching theme, main categories and sub-categories

Overarching theme	“The being and becoming of a mother – equanimity in the transition to motherhood”		
Main categories	Sub-categories		
**Inner resources**	Trusting the process of pregnancy	Being your own best friend	Gatekeeping the mind
**Experiencing trust during the transition**	Having a safe environment	Sharing the transition	Sense of fairness and equity
**Belonging to a supportive and caring community**	Peer support	Caring and supportive network

i) Inner resources
  An individual’s inner resources in coping with the transition to motherhood were described by health care professionals as important for mental wellbeing during pregnancy. These inner resources included the sub-categories: trusting the process of pregnancy, being your own best friend, and gatekeeping the mind. The informants described these inner resources as women’s abilities that could be reinforced and thus support in the transition to motherhood.
  *Trusting the process of pregnancy* was described as letting go and adapting to the uncertainty of pregnancy. For example, being able to recognise that pregnancy is a limited time period and having a mindset of acceptance and flexibility during this period; trusting that present mood and feelings will pass and not being too alarmed in the moment. Professionals also talked about acceptance in relation to the built-in uncertainty of ‘“horticultural time”’; i.e., the birth, could happen at any time within a specific time frame at the end of pregnancy; and various other associated changes (i.e., bodily, relational, or psychological changes during pregnancy). Adjusting expectations of pregnancy and future motherhood with the present moment was also described as a feature of ‘trusting the process’ of pregnancy.

“One can sometimes choose how to… relate to things that happen. Maybe an acceptance that ‘yes, it is difficult, it is hard’” Informant 4

*Being your own best friend* was described by healthcare professionals as important for mental wellbeing during pregnancy. This included having the ability to self-soothe, be kind to yourself and being able to accommodate to one’s needs. That is, being able to identify activities and situations that can enhance wellbeing by, e.g., increasing positive feelings and having healthy structures for sleep, food, physical activity as well as leaving space for enjoyable activities. Being your own best friend was described as helping pregnant women to nurture themselves as well as reach out and get help by remaining attentive to their needs, establishing healthy boundaries and finding a “good enough” level regarding what to accomplish. In other words, being able to take care of yourself and calibrate both inner demands as well as the demands perceived by others.

*”the responsibility to satisfy the needs of the child does not automatically exclude meeting your own needs” Informant 6*.

*Gatekeeping the mind* was described by healthcare professionals as an important inner resource for the wellbeing of pregnant women. For example, choosing when to think about certain things to avoid being overwhelmed.

*”… it is that compass that is so easy to lose*,* and be overwhelmed by advice and by everybody*,* and by all homepages you visit*,* and all the influencers in glossy journals. And*,* yes*,* you want to keep a hold of that compass.” Informant 5*.

Healthcare professionals described a significant difference in the information flow of today with that before the of use of smartphones. They underlined the increased need for a gatekeeping ability to be able to experience equanimity. Gatekeeping the mind was described as the ability to navigate the overwhelming flow of information (e.g., from social media, magazines, and news) in today´s society.

ii) *Experiencing trust during the transition*
  The healthcare professionals described mental wellbeing during pregnancy as having the possibility to experience safety and trust during the transition to motherhood. The informants highlighted several preconditions that were essential to be able to experience safety and trust e.g., access to a physically safe environment. Furthermore, sharing the transition to motherhood with a partner or another close relationship as well as having a sense of fairness and equality in the changing partnership of parenthood, e.g., in relation to the division of unpaid housework, was described as important.
  *Having a safe environment* was described as ensuring the basic habitual and financial needs fundamental for mental wellbeing according to healthcare professionals. The fundamentals for a safe environment were a stable economy and shelter that met the basic requirements for putting food on the table and enabling the basic care of a newborn. Paid parental leave was also described as an important basis for mental wellbeing during the transition to motherhood.

*“Economy*,* I think*,* is about feeling secure in the finances you have. That you don’t have to worry about how you’ll afford this and that next month*,* and that it was much more expensive than we thought to have a small child.” Informant 1*.

*Sharing the transition* was described as the importance of having a partner, a close friend, or a family member with whom one could share both the experience and responsibility. The informants emphasised the importance of not being left alone during pregnancy and having someone to resonate with and calibrate towards during the transition to motherhood. The qualities of such a sharing relationship were identified by professionals as having the sense of being cared for, of being able to communicate, prepare, and plan for the future as well as the ability to calibrate expectations. Building a trusting and assertive relationship sharing both joy and happiness as well as struggles was highlighted as important during pregnancy and early motherhood.

*“support from the partner is extremely important… women will get through pregnancy*,* however*,* [those who are] giving birth on their own are definitely more vulnerable..women who do have a partner*,* who is positive about the future child*,*. is starting to re-adjust and to have expectations… how they suddenly become aware and be more present*,* ask questions*,* show care of the woman in different ways” Informant 2*.

*A sense of fairness and equity* was also described as an important ingredient in experiencing trust during pregnancy. For example, according to healthcare professionals a sense of fair distribution of chores in the family organisation and equal opportunities in work life were considered important. The sense of fairness and equity was thought to be deeply connected to expectations of pregnancy and motherhood. Informants described the disparity between expectations on pregnancy and motherhood and how this reality negatively influenced mental wellbeing during pregnancy.

*“it is really sad to hear how unfair people are living [in terms of dividing domestic chores and child rearing]… still. I think this can be a huge contributing cause as to why mothers are feeling bad.” Informant 3*.

iii) Belonging to a supportive and caring community
  The healthcare professionals described the network surrounding the woman as important for mental wellbeing during pregnancy. Belonging to a supportive and caring community was described as protecting from feeling alone and lonely. Furthermore, it was described as offering opportunities to recognise oneself in others, share experiences with peers, as well as to receive care and support when needed.
  *Peer support* was described as important for pregnant women and new mothers. Health care professionals perceived that pregnant woman, and especially a new mother, needs to surround herself with an environment in which she feels safe and can calibrate her motherhood. Professionals expressed the benefit of authentic role models depicting real life parenting, and peer support groups where women can exchange information and experiences.

*“gathering in groups of very different women [with different experiences of pregnancy]. So that women who are afraid can meet unafraid women. And to see that it is not only me who is living in this reality… there are other women*,* who think differently.*

*This meeting the social part… it is invaluable to have the physical parental educations. To strengthen the mental wellbeing of pregnant women.” Informant 3*.

Peer support was also described by the informants as offering a sense of belonging to a community that can guide the transition to motherhood. The possibility of sharing thoughts and seeing that others have the same thoughts was perceived to help to normalise mixed feelings about pregnancy and parenthood.

The healthcare professionals underlined the importance of real-life peer support e.g., parental groups as an antidote to the potentially negative impact of social media. The informants described that social media could contribute to unrealistic expectations of pregnancy and motherhood illustrating either unrealistic ideals or worse-case scenarios.

Another important function of peer support was described as being able to see other parents and how they interact with their child. Accordingly, peer support groups were described as having the dual function of both providing a sense of belonging to a community and a support network.

*Having a caring and supportive network* was described by the informants as building on both the quality of the relationships and the access to social support on mental, emotional and practical levels. The mental support was described by professionals as having a trusting and caring relationship. Practical support was expressed by the informants as building a safety net around the pregnant woman and potentially around the growing family unit, making it less vulnerable and relieving stress by having somebody taking the baby for a walk, for example, to allow the new parents to rest.

The informants expressed that individual support given by midwives and child healthcare nurses can sometimes be the only source of support for a pregnant woman or new mother. Sometimes this is the only place she has to turn to for advice or a second opinion depending on what other social networks surround the woman.

*”I believe in [meeting] the midwife in real life. It has become very popular to have online midwives… it can be a great complementary support. However*,* for anxious mothers-to-be*,* it is definitely good to meet in real life where you can meet a real person …. someone who will be there and follow you through the whole transition.” Informant 2*.

## Discussion

This study aimed to explore mental wellbeing during pregnancy and the post-partum period from a professional healthcare perspective. According to findings, perinatal mental wellbeing can be described as ‘*The being and becoming of a mother– equanimity in the transition to motherhood’* that is, a process where women strive towards finding balance during a life-changing period. Findings also included factors that were perceived to support a healthy transition to motherhood i.e., inner resources such as self-kindness, experiencing trust during the transition and having access to a caring and supportive network.

The findings correspond with existing theory and definitions of mental wellbeing. For example, perceiving mental wellbeing as a balancing act within the overarching theme is in line with Dodge et al. who propose equilibrium as central in their definition of mental wellbeing [40]. They illustrate mental wellbeing as balancing on a seesaw, with coping resources on one side and life challenges on the other side. Equilibrium, or balance, is met when individuals have the psychological, social, and physical resources they need to meet a particular challenge. When individuals have more challenges than resources, the seesaw dips, as does mental wellbeing, and vice-versa. Furthermore, Ryff’s six-dimension theory of psychological wellbeing [41] proposes that self-acceptance, positive relations, autonomy (e.g., resisting social pressures), environmental mastery (e.g., being in contexts that fit one’s values), purpose in life and personal growth, are all factors that together contribute to mental wellbeing. Also, the theory emphasizes that psychological well-being is not just about “feeling happy” but involves achieving a balanced state influenced by both challenging and rewarding life events. Our findings of *Inner resources* are in line with several of these dimensions described, for example, *Trusting the process of pregnancy* which included acceptance (of self and the situation at hand). In addition, the categories ‘*Experiencing trust during the transition’* and ‘*Belonging to a supportive and caring community’* support Ryff’s theory highlighting the importance of positive relations in mental wellbeing. Thus, highlighting both psychological and social aspects of mental wellbeing, supporting the idea that mental wellbeing is multi-dimensional, and that mental health promotion needs to consider individual- community and societal level factors. Our findings may advance the understanding of mental wellbeing by contributing knowledge on what encompasses mental wellbeing during the perinatal period per se.

Informants talked about equanimity in terms of calibrating expectations (of pregnancy and motherhood) with real life, which resonates with previous research. In their grounded theory study, Darvil et al. interviewed new mothers and found that supporting women in forming realistic expectations already in early pregnancy and motherhood was important [32]. In another interview study, pregnant women described how experiences such as sense of self-doubt, loss of control of emotions, guilt and stigma, were all real experiences but perceived to be at odds with how pregnancy should feel [42]. A systematic review and meta-ethnographic synthesis of young women’s perceptions of pregnancy identified social judgement and stigmatisation as key factors affecting mental health during pregnancy and motherhood [43]. Specifically, young women (< 20 years) felt that they had to represent themselves as ‘good mothers’ and believed that mental health problems contradicted the narratives to which they were expected to conform [43]. Another qualitative review showed that women who experienced distress during pregnancy interpreted this experience as deviant and deemed their future selves as inadequate mothers [42]. Hence, supporting women in calibrating their expectations of pregnancy and motherhood, presenting diverse and realistic pregnancy experiences, is important in perinatal mental health promotion.

The findings proposed several inner resources (e.g., self-kindness, acceptance) that health care professionals perceived to support a healthy transition to motherhood. Previous intervention research has been shown to increase mental wellbeing during pregnancy through resilience [17, 24]. Mindfulness-based interventions, for example, have shown promising effects on empowerment, positive affect and self-compassion as well as effects on decreased anxiety, depressive symptoms and perceived stress among pregnant women [19, 23]. An integral part of these interventions is to build trust in the process of pregnancy and promote self-compassion (i.e., being your own best friend) to enhance mental wellbeing. Altogether, our findings are in accordance with this body of research demonstrating that inner resources are key factors for the promotion of perinatal mental wellbeing.

Another key finding was the importance of experiencing trust and sharing the transition to motherhood with a partner or other close relationship. Similarly, in an interview study with women on perinatal mental health, sharing their experiences was described as something offering solace during pregnancy and after birth [44]. Moreover, the importance of positive relationships for mental wellbeing has been described in other studies where enhancing emotional intimacy and self-compassion among expectant couples has been described as important for those with a history of adversity [45, 46]. In addition, a study that explored experiences of “solo-mothers” on creating a “solo-family” described the importance of grandparents as co-parents to be able to share experiences [47].

Furthermore, experiencing trust in terms of equity was also described in our data. Similarly, in an interview study with new mothers on maternal mental health, the need to share parenting responsibilities, decision-making, financial and practical arrangements and to feel cared for, were expressed to be important [3]. Reports utilizing group-based trajectory modelling of data from the Swedish Longitudinal Occupational Survey of Health (SLOSH 2008–2014) showed that women generally worked longer hours and spent more time doing unpaid work compared with men, which contributed to an increased risk of depression [48]. Altogether, these data confirm our findings that gender equity is an important aspect to consider in perinatal mental health promotion.

Furthermore, our findings emphasize the importance of belonging to a caring and supportive community during pregnancy and after birth is supported by previous research [32, 49–51]. For example, Bedaso et al. found that low social support was associated with an increased risk of developing mental health problems such as depression and anxiety during pregnancy [49]. Similarly, Battulga el al. found an association between functional social support and subjective wellbeing during pregnancy [51]. Finally, Corno et al. confirmed the importance of perceived social support but distinguished between different sources of support e.g., support from family (preventative effect on psychological distress) and support from friends (promoting wellbeing) [50]. In addition, the Swedish National Board for Health and Welfare has underlined the importance for health care organizations to prioritise parental group activities to provide peer support for pregnant women and new mothers [52]. Thus, research suggests that functional and social support including the provision of emotional, practical and informational support is vital for mental wellbeing during pregnancy.

Finally, our findings proposed a potentially negative impact of the social media community during both pregnancy and after birth, e.g., through unrealistic ideals of how pregnancy should be like. However, Corno et al. argues that there is potential to use social media platforms to establish social support when there are limitations for face-to-face interactions such as during the pandemic [50]. Thus, leading to more women feeling less alienated in their experiences and promoting mental wellbeing during pregnancy. A cross-sectional survey conducted in China during the COVID-19 pandemic showed that the frequency of social media use for finding health information was indirectly associated with higher levels of worry and depression [53]. Although cross-country comparisons can be difficult regarding mental wellbeing, Wang et al. argues that interventions are needed to empower pregnant women with the skills needed to identify credible sources to obtain health information, to provide thoughtful consideration of the veracity and quality of health information, and to process the information in an objective manner [53].

### Implications for practice

Our findings highlight mental health promoting factors during the perinatal period existing on multiple levels (e.g., societal and structural support). Thus, the findings propose a comprehensive approach to mental health promotion during the perinatal period. This is in line with socio-ecological perspectives on health and wellbeing as illustrated by e.g., Wadepuhl et al.’s tentative model of perinatal wellbeing [54]. Our findings have implications on the macro level by suggesting the importance of e.g., parental leave and childcare to promote mental wellbeing during pregnancy on a societal level. Hence, creating societal structures enabling pregnant women to gain financial security (through e.g., parental allowance) and family’s opportunities to divide unpaid work equally, could offer optimal conditions for perinatal mental wellbeing. Furthermore, our findings showed the growing influence of social media on pregnancy and motherhood through e.g., unrealistic ideals, excess health information. These findings have implications on several levels e.g., the need for digital communities presenting realistic and diverse pregnancy experiences, health care support for women navigating health information (gatekeeping) or organized in-person peer groups offered within maternity- and child health care.

Our findings have implications for individual-level interventions by suggesting several inner resources with potential to build resilience during the perinatal period. Specifically, the findings indicate a need for mental health promotion within women’s health care to target abilities for mental wellbeing such as acceptance, trusting the process of pregnancy and self-kindness. Finally, developing accessible digital interventions (e.g., mobile applications) to promote self-care practices could be a cost-effective approach to foster inner resources during the perinatal period as identified in these interviews. The current study was an initial explorative step in the development of a digital mental health promotion intervention targeting perinatal women (forthcoming manuscript). For this work, the findings suggest not only key inner resources to be promoted, but also the importance of fostering healthy close relationships and opportunities for being part of safe communities with other pregnant women and new mothers.

### Strengths and limitations

Trustworthiness in terms of transferability and credibility was increased through the use of a purposive sampling strategy. Specifically, multiple professions and perspectives throughout perinatal health care were included to gain rich data with maximum variation. Thus, informants with experience and expertise on mental health during pregnancy and early motherhood were recruited. The health care professional perspective of the study can also be seen as a potential limitation (rather than interviewing e.g., pregnant women or partners). However, by employing a professional perspective, informants could talk about mental wellbeing during pregnancy from an ‘outsider’ perspective based on their clinical experience of meeting women from socioeconomic diverse areas. Also, the findings are supported by previous qualitative research with pregnant women and new mothers [3, 32]. Trustworthiness in terms of credibility was further increased through investigator triangulation in the analysis where three researchers with varied experience and perspectives conducted the analysis. Dependability was increased using an interview-guide and field notes throughout data collection. A potential challenge could have been that most interviews were conducted over video call. However, we experienced that the informants were able to sufficiently reflect on the questions at hand which we perceived contributed to gain rich data. In addition, a strength of using videocall interviews was that we could recruit informants throughout Sweden including rural and urban locations and different socio-economic areas. Finally, regarding transferability, level of economic development, and different cultures especially with respect to gender relations and overarching political and institutional structures need to be considered. The study was conducted in a developed country with a governmental paid parental leave system comparable to Norway, Austria, Germany, Japan, Canada, Italy and many more OECD countries.

## Conclusion

Mental wellbeing during the transition to motherhood can be supported by increasing the opportunities available to pregnant women and building resources for equanimity among pregnant women. Findings emphasise the need to target health promoting factors on multiple levels including strengthening the inner resources of the individual but also building social support structures and communities. The saying “It takes a village to raise a child and a network to create a mother” supports our results. A close cooperation within the chain of care in the healthcare organisation is vital to identify women in need of extra support and to work together to promote mental wellbeing during early pregnancy.

## Supplementary Information

Supplementary Material 1.

Supplementary Material 2.

## Data Availability

The data analysed are not publicly available as individual privacy could be compromised but are available from the corresponding author on reasonable request.
