# Supplementary material for: Mental wellbeing during pregnancy and the transition to motherhood: an explorative study through the lens of healthcare professionals
Source: BMC Pregnancy Childbirth. 2025 Aug 1;25:803. doi: 10.1186/s12884-025-07894-5 (PMC12317509; doi:10.1186/s12884-025-07894-5)
Supplement: Supplementary file 2 — Supplementary Material 2. [file 12884_2025_7894_MOESM2_ESM.docx]

| **Supplementary material**  **Supplementary material 1: Examples of the data analysis process from unit of analysis to main category** | | | | |
| --- | --- | --- | --- | --- |
| **Unit of analysis** | **Code** | **Subcategory** | **Generic category** | **Main category** |
| “one can choose sometimes how to… relate to things that happen. Maybe an acceptance that ‘yes it is difficult, it is hard’”  (informant 4) | Acceptance | Acceptance | Trusting the process of pregnancy | Inner resources |
| ”a trust to oneself that ’yes, I can birth a child, others have done it. My body will also be able to do it’”. (informant 1) | Self-efficacy | Self-efficacy | Trusting the process of pregnancy | Inner resources |
| ”the responsibility to satisfy the needs of the child does not automatically exclude your own needs to be met” (informant 4) | Own needs | Balancing needs | Being your own best friend | Inner resources |
| ”and it is that compass that is so easy to loose, and be overwhelmed by all advice and everybody, all homepages you visit. And all influencers in glossy journals. And, yes, you want to keep hold of that compass.” (informant 5) | Feeling overwhelmed | Gate-keeping | Gate-keeping the mind | Inner resources |
